# Cardiovascular disease relates to intestinal uptake of *p*-cresol in patients with chronic kidney disease

**DOI:** 10.1186/1471-2369-15-87

**Published:** 2014-06-09

**Authors:** Ruben Poesen, Liesbeth Viaene, Kristin Verbeke, Patrick Augustijns, Bert Bammens, Kathleen Claes, Dirk Kuypers, Pieter Evenepoel, Björn Meijers

**Affiliations:** 1 Department of Microbiology and Immunology, Division of Nephrology, University Hospitals Leuven, B-3000, Leuven, Belgium; 2 Translational Research for Gastrointestinal Disorders (Targid) and Leuven Food Science and Nutrition Research Centre (LFoRCe), University of Leuven, B-3000 Leuven, Belgium; 3 Department of Pharmaceutical and Pharmacological Sciences, Drug Delivery and Disposition, University of Leuven, B-3000 Leuven, Belgium; 4 Division of Internal Medicine, Department of Nephrology, University Hospitals Leuven, Herestraat 49, B-3000 Leuven, Belgium

**Keywords:** Cardiovascular disease, Gut, *P*-cresyl sulfate

## Abstract

**Background:**

Serum *p*-cresyl sulfate (PCS) associates with cardiovascular disease in patients with chronic kidney disease. PCS concentrations are determined by intestinal uptake of *p*-cresol, human metabolism to PCS and renal clearance. Whether intestinal uptake of *p*-cresol itself is directly associated with cardiovascular disease in patients with renal dysfunction has not been studied to date.

**Methods:**

We performed a prospective study in patients with chronic kidney disease stage 1 – 5 (NCT00441623). Intestinal uptake of *p*-cresol, under steady state conditions, was estimated from 24 h urinary excretion of PCS. Primary endpoint was time to first cardiovascular event, i.e., cardiac death, myocardial infarction/ischemia, ventricular arrhythmia, cardiovascular surgery, ischemic stroke or symptomatic peripheral arterial disease. Statistical analysis was done using Kaplan-Meier estimates and Cox proportional hazard analyses.

**Results:**

In a cohort of 200 patients, median 24 h urinary excretion of PCS amounted to 457.47 μmol (IQR 252.68 – 697.17). After a median follow-up of 52 months, 25 patients reached the primary endpoint (tertile 1/2/3: 5/6/14 events, log rank *P* 0.037). Higher urinary excretion of PCS was directly associated with cardiovascular events (univariate hazard ratio per 100 μmol increase: 1.112, *P* 0.002). In multivariate analysis, urinary excretion of PCS remained a predictor of cardiovascular events, independent of eGFR (hazard ratio 1.120, *P* 0.002).

**Conclusions:**

In patients with chronic kidney disease, intestinal uptake of *p*-cresol associates with cardiovascular disease independent of renal function. The intestinal generation and absorption of *p*-cresol may be therapeutic targets to reduce cardiovascular disease risk in patients with renal dysfunction.

## Background

Chronic kidney disease (CKD) is accompanied by the accumulation of so-called uremic retention solutes [1]. There is mounting evidence that the colonic microbial metabolism contributes substantially to these solutes with *p*-cresyl sulfate (PCS) being among the most discriminating biomarkers of uremia [2]. PCS has been linked to cardiovascular disease [3-5], overall mortality [6,7] and progression of CKD [8].

Serum concentrations of PCS result from the combined actions of intestinal uptake of *p*-cresol, metabolism (i.e., sulfate conjugation) to PCS, and eventually, renal excretion of PCS [9]. Renal dysfunction causes PCS to accumulate, mainly due to diminished active tubular secretion [10], and higher serum concentrations of PCS are related to cardiovascular disease in patients with CKD [3,7]. Until recently, it was assumed that kidney function was the main determinant of serum PCS. However, after adjustment for renal function loss, individual serum concentrations of PCS are still widely dispersed [3,7,11]. Intriguingly, our group identified intestinal uptake of *p*-cresol to be a main determinant of this interindividual variability [12]. Total intestinal uptake of *p*-cresol can be considered a composite of intestinal generation (i.e., colonic microbial fermentation of tyrosine) and subsequent intestinal disposition, although mechanisms governing both processes are largely unexplored.

Whether intestinal uptake of *p*-cresol itself, independent of kidney function, is associated with cardiovascular disease has not been studied to date.

We aimed to explore potential clinical determinants of the intestinal uptake of *p*-cresol and to study in depth its relationship with cardiovascular disease in patients at different stages of CKD.

## Methods

### Study population

This is a secondary analysis of the Leuven mild to moderate CKD cohort (clinicaltrials.gov NCT00441623) [3]. Prevalent CKD patients, followed at the nephrology outpatient clinic of the University Hospitals Leuven, 18 year of age or older and able to provide consent, were eligible for inclusion. Patients were screened between November 2005 and September 2006. Data on baseline demographics were collected at time of informed consent. The study was performed according to the Declaration of Helsinki and approved by the ethics committee of the University Hospitals Leuven. Informed consent was obtained from all patients.

### Biochemical measurements

At inclusion, blood was taken by venous puncture for measurement of hemoglobin (g/dl), albumin (g/l), C-reactive protein (mg/l), cholesterol (mg/dl), calcium (mg/dl), phosphate (mg/dl), biointact parathyroid hormone (PTH) (ng/l), creatinine (mg/dl), blood urea nitrogen (g/dl) and PCS (μM). Hemoglobin, albumin, C-reactive protein, cholesterol, calcium, phosphate, biointact PTH, creatinine and blood urea nitrogen were all measured using standard laboratory techniques. The eGFR was calculated using the CKD-EPI equation [13]. PCS was quantified using a dedicated high-performance liquid chromatography (HPLC) method as described previously [14]. Assuming steady state conditions, 24 hour intestinal uptake of *p*-cresol was estimated from 24 hour urinary excretion of PCS. 24 hour urinary collections were sampled when available at time of inclusion. Completeness was assessed using 24 hour urinary creatinine excretion. Collections were considered complete when 24 hour urinary creatinine excretion was within 2 standard deviations of the mean creatinine excretion for the geographical region of this study, derived from the INTERSALT study [15]. Protein intake was calculated according to the formula of Maroni *et al.* by using 24 hour urinary urea nitrogen excretion and body weight [16,17].

### Endpoint evaluation

After inclusion, patients were prospectively followed at the nephrology outpatient clinic at 3- to 6-month intervals until December 31, 2010. Predefined endpoints were prospectively recorded and coded, blinded from clinical and biochemical data. The primary endpoint (cardiovascular event during follow-up) was a composite of death from cardiac causes, non-lethal myocardial infarction, myocardial ischemia, coronary intervention, ischemic stroke, or peripheral vascular disease, whichever occurred first. Only one event per subject was included in the analysis. After review of available information, cause of death was classified as either cardiovascular, infectious, malignancy, or other. Cardiovascular deaths included fatal myocardial infarction, sudden death, and death due to congestive heart failure. Cases of unobserved sudden death were considered cardiovascular death only when other potential causes could be excluded. Otherwise, they were classified as other cause of death. Out-of-hospital deaths were coded after consultation of the general practitioner. Non-lethal cardiovascular events included myocardial infarction, diagnosed based on elevated levels of cardiac enzymes and/or typical electrocardiography changes, myocardial ischemia with typical electrocardiography changes without elevated cardiac enzymes, coronary intervention (thrombolysis, percutaneous coronary intervention, or coronary artery bypass grafting), and ventricular arrhythmia. Ischemic stroke was defined as a neurologic deficit lasting more than 24 hours. Hemorrhagic stroke was excluded from the primary endpoint. Peripheral vascular disease included new-onset ischemic pain in the lower limbs, with abnormal ankle brachial pressure index or radiologic evidence of peripheral vascular disease, new-onset ischemic necrotic lesions, or surgical arterial intervention. Secondary endpoints included overall mortality and progression of renal disease, defined as progression to renal replacement therapy and/or doubling of serum creatinine during follow-up.

### Statistical analysis

Data are expressed as mean (standard deviation) for normally distributed variables or median (interquartile range (IQR)) for non-normally distributed variables. Differences between baseline variables according to tertiles of 24 h urinary excretion of PCS were tested using parametric ANOVA, Kruskal-Wallis or chi-squared test as appropriate. Correlations between 24 h urinary excretion of PCS and other variables were calculated by Spearman’s rank correlation coefficients. To identify independent determinants of 24 h urinary excretion of PCS, multivariate linear regression analysis was performed. Relevant demographic (i.e., age, gender, presence of diabetes mellitus, smoking status, body mass index) and biochemical (i.e., hemoglobin, C-reactive protein (Ln), albumin, eGFR, 24 hour proteinuria (Ln), 24 h protein intake) parameters were first subjected to a backward elimination procedure on *P* < 0.2, with subsequently, a final backward elimination step on *P* < 0.05. The Kaplan-Meier method was used to estimate cumulative incidence of the endpoint with the log rank test to compare differences between tertiles of 24 h urinary excretion of PCS. Time to first event analysis was performed using Cox proportional hazards analysis. Besides 24 h urinary excretion of PCS, other relevant demographic (i.e., age, gender, presence of diabetes mellitus, prior cardiovascular disease, smoking status, body mass index, systolic blood pressure, use of statin therapy, use of angiotensin converting enzyme inhitor/receptor blocker therapy) and biochemical (i.e., hemoglobin, albumin, C-reactive protein (Ln), cholesterol, calcium, phosphate, PTH (Ln), creatinine, eGFR, 24 h proteinuria (Ln)) variables were selected from univariate analysis (*P* < 0.2). With this subset of variables different multivariate models were built and subjected to backward elimination (*P* < 0.05). Due to the limited number of events (*n* = 25), no more than three variables (24 h urinary excretion of PCS with two other variables) were included in each model. To test the proportionality assumption, each model was tested against log(time). In case the proportionality assumption was violated, time-dependent covariates were entered into the model. For both Kaplan-Meier and Cox proportional hazard analysis of the primary endpoint, data were censored at start of renal replacement therapy, death other than cardiovascular, loss to follow-up, or at the end of the study observation period. With respect to the secondary endpoints, data were censored at start of renal replacement therapy (for overall mortality), death (for renal disease progression), loss to follow-up, or at study end. For all statistical analysis, *P*-values less than 0.05 were considered significant. All statistical analyses were performed using SAS (version 9.3, the SAS institute, Cary, NC, USA).

## Results

### Inclusion and baseline characteristics

**Figure 1:**
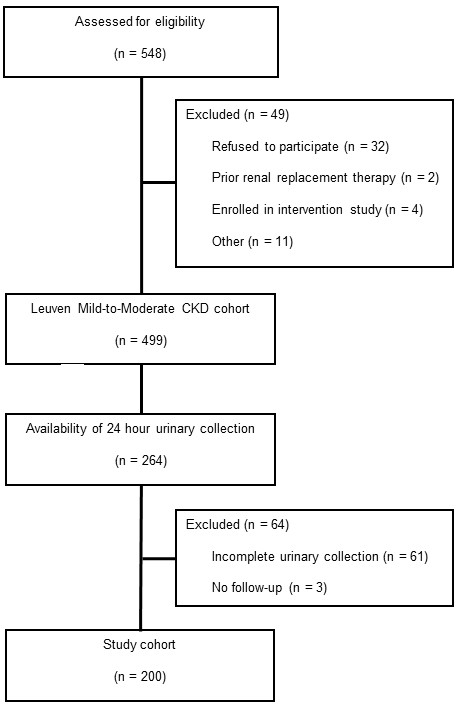
Patient inclusion, flow chart demonstrating patient screening and inclusion.

Between November 2005 and September 2006, 548 prevalent patients with CKD K/DOQI stage 1 – 5, followed at the nephrology outpatient clinic of the University Hospitals Leuven, Belgium were found eligible to be enrolled in the Leuven mild to moderate CKD study. 499 patients providing informed consent were included [3]. Of these, urinary collections were available for 264 patients and considered complete for 203 patients. No further follow-up was scheduled for 3 patients, making a total of 200 patients to be included in the prospective cohort study (Figure 1). Baseline characteristics of the study population are presented in Table 1. Glomerular disease was the most prevalent underlying renal disease (37.5%), followed by undetermined cause (35.5%), vascular disease (9.0%), and diabetic nephropathy (6.5%). Apart from a small, but significant age (median 4 years younger, *P* 0.01) and systolic blood pressure (median 3 mmHg lower, *P* 0.05) difference, we observed no significant differences between the current study population and the original patient cohort.

**Table 1:** Baseline characteristics of study population

**Variable**	**Overall (n = 200)**	**24 h urinary excretion of *p*-cresyl sulfate**			***P***
		**Tertile 1 (n = 66)**	**Tertile 2 (n = 67)**	**Tertile 3 (n = 67)**	
Age (yr)	60 (48–72)	58 (47 – 70)	59 (44–72)	64 (50 – 73)	0.15
Gender: male/female (%)	119/81 (59.5/40.5)	34/32 (51.5/48.5)	41/26 (61.2/38.8)	44/23 (65.7/34.3)	0.10
Prior CVD: yes/no (%)	53/147 (26.5/73.5)	13/53 (19.7/80.3)	18/49 (26.9/73.1)	22/45 (32.8/67.2)	0.09
Diabetes: yes/no (%)	31/169 (15.5/84.5)	6/60 (9.1/90.9)	7/60 (10.4/89.6)	18/49 (26.9/73.1)	**0.005**
Current smoker: yes/no (%)	34/166 (17/83)	14/52 (21.2/78.8)	7/60 (10.4/89.6)	13/54 (19.4/80.6)	0.79
Body mass index (kg/m^2^)	25.67 (22.99 – 28.67)	24.91 (23.31 – 29.63)	25.43 (22.65 – 28.60)	26.21 (23.12 – 29.06)	0.80
Systolic blood pressure (mmHg)	132 (120 – 150)	130 (120 – 150)	134 (120 – 150)	132 (120 – 150)	0.72
Diastolic blood pressure (mmHg)	80 (70 – 85)	80 (70 – 85)	80 (70 – 85)	80 (70 – 85)	0.82
Hemoglobin (g/dl)	13.5 (1.7)	13.5 (1.7)	13.6 (1.8)	13.3 (1.8)	0.44
Albumin (g/l)	45.1 (42.0 – 46.8)	44.8 (41.9 – 46.3)	45.8 (43.3 – 47.7)	44.8 (41.4 – 46.2)	0.06
C-reactive protein (mg/l)	2 (1 – 5)	2 (1 – 6)	1 (1 – 4)	2 (1 – 6)	0.06
Cholesterol (mg/dl)	181 (153 – 205)	185 (152 – 209)	183 (159 – 200)	166 (151 – 205)	0.35
LDL (mg/dl)	83 (66 – 107)	77 (61 – 112)	90 (70 – 113)	81 (66 – 100)	0.23
Calcium (mg/dl)	9.5 (9.3 – 9.9)	9.6 (9.3 – 9.9)	9.4 (9.1 – 9.8)	9.5 (9.2 – 9.8)	0.68
Phosphate (mg/dl)	3.4 (2.9 -3.8)	3.3 (2.9 – 3.8)	3.4 (2.9 – 4)	3.4 (2.9 – 3.8)	0.88
Bicarbonate (mmol/l)	24.6 (2.9)	24.4 (2.9)	24.8 (2.7)	24.5 (3.1)	0.74
Creatinine (mg/dl)	1.82 (1.33 – 2.53)	1.69 (1.13 – 2.50)	1.86 (1.34 – 2.77)	2.01 (1.42 – 2.4)	0.31
eGFR (ml/min per 1.73 m^2^)	33.4 (22.8 – 55.5)	37.9 (24.7 – 61.2)	31.5 (22.8 – 61.0)	32.0 (21.6 – 50.4)	0.44
24 hour proteinuria (g)	0.307 (0.108 – 1.128)	0.243 (0.098 – 1.128)	0.256 (0.091 – 0.891)	0.429 (0.192 – 1.380)	**0.03**
Therapy with ACEI/ARB: yes/no (%)	155/45 (22.5/77.5)	53/13 (80.3/19.7)	50/17 (74.6/25.4)	52/15 (77.6/22.4)	0.71
Therapy with statins: yes/no (%)	113/87 (56.5/43.5)	33/33 (50/50)	36/31 (53.7/46.3)	44/23 (65.7/34.3)	0.07
24 h urinary excretion of PCS (μmol)	457.47 (252.68 – 697.17)	183.76 (51.14 – 250.03)	454.11 (375.92 - 515.67)	822.02 (694.71 – 1119.62)	**< 0.0001**
Serum PCS (μM)	46.48 (20.40 – 94.36)	16.91 (5.71 – 38.41)	46.57 (26.49-89.60)	79.18 (52.58 –152.34)	**< 0.0001**

Data are expressed as mean (SD) or median (IQR), as appropriate. Differences between tertiles were tested using parametric ANOVA, Kruskal-Wallis of chi-squared test, as appropriate. CVD, cardiovascular disease; LDL, low-density lipoprotein; eGFR, estimated glomerular filtration rate; ACEI, angiotensin-converting enzyme inhibitor; ARB, angiotensin receptor blocker; PCS, *p*-cresyl sulfate.

### Correlations of 24 h urinary excretion of PCS

24 h urinary excretion of PCS amounted to a median of 457.47 μmol (IQR 252.68 – 697.17). There was a moderate correlation between 24 h urinary excretion of PCS and serum PCS (Spearman’s rank correlation *ρ* 0.64, *P* < 0.0001) (Table 2). In addition, there were significant, though minor correlations between 24 h urinary excretion of PCS and 24 h intake of protein (*ρ* 0.30, *P* < 0.0001) as well as presence of diabetes mellitus (*ρ* 0.18, *P* < 0.0001). Patients with diabetes mellitus had a significantly higher 24 h urinary excretion of PCS than patients without diabetes mellitus (median 668.20 μmol (IQR 403.05 – 1161.10) vs. 427.02 μmol (IQR 681.36 – 238.37), *P* 0.009). Although there was a trend of increasing 24u urinary excretion of PCS with higher 24 h proteinuria, higher age, prior cardiovascular disease, and lower eGFR, these correlations did not reach significance.

**Table 2:** **Spearman’s rank correlation between 24 h urinary excretion of *p*-cresyl sulfate and baseline characteristics**

**Variable**	***ρ***	***P***
Age	0.13	0.07
Gender (female vs. male)	−0.08	0.25
Prior CVD	0.12	0.09
Diabetes mellitus	0.18	**0.009**
Current smoker	0.0007	0.99
Body mass index	0.01	0.84
Systolic blood pressure	0.06	0.38
Diastolic blood pressure	0.03	0.64
Hemoglobin	−0.07	0.35
Albumin	−0.03	0.70
C-reactive protein	−0.05	0.47
Cholesterol	−0.06	0.43
LDL	0.03	0.68
Calcium	−0.03	0.71
Phosphate	0.03	0.65
Parathormone	0.09	0.20
Bicarbonate	0.02	0.82
Creatinine	0.11	0.11
eGFR	−0.10	0.15
24 h proteinuria	0.14	0.05
24u protein intake	0.30	**< 0.0001**
Therapy with ACEI/ARB	−0.008	0.91
Therapy with statin	0.13	0.06
Serum PCS	0.64	**< 0.0001**

CVD, cardiovascular disease; LDL, low-density lipoprotein; eGFR, estimated glomerular filtration rate; ACEI, angiotensin-converting enzyme inhibitor; ARB, angiotensin receptor blocker; PCS, *p*-cresyl sulfate.

In multivariate analysis, independent determinants of 24 h urinary excretion of PCS were age (*β* 4.30, *P* 0.03), presence of diabetes mellitus (*β* 202.67, *P* 0.01), body mass index (*β* -19.59, *P* 0.003), hemoglobin (*β* - 41.01, *P* 0.01) and 24 h protein intake (*β* 9.21, *P* < 0.0001) (Model *R*^*2*^ 0.22) (Table 3).

**Table 3:** **Multivariate regression analysis: Factors associated with 24 h urinary excretion of *p*-cresyl sulfate**

**Variable**	**Unit**	***β***	***P***
Age	year	4.30	0.03
Presence of diabetes mellitus	yes vs no	202.67	0.01
Body mass index	kg/m^2^	−19.59	0.003
Hemoglobin	g/dl	−41.01	0.01
24 h protein intake	g	9.21	< 0.0001
		Model *R*^*2*^	0.22

### Event analysis

**Figure 2:**
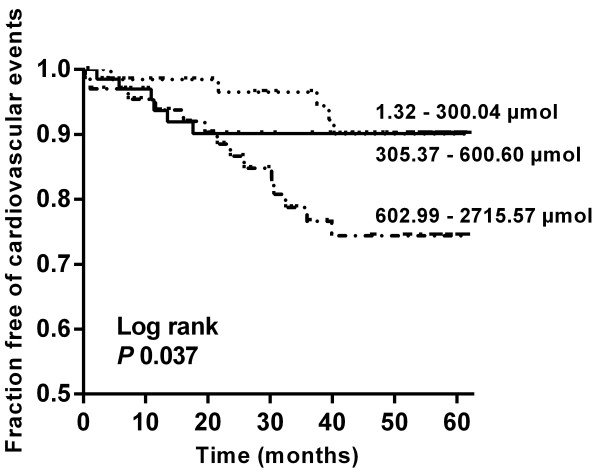
**Kaplan-Meier curve of time to first cardiovascular event.** Tertiles of 24 h urinary excretion of *p*-cresyl sulfate. Tertile 1 to 3: 5, 6 and 14 events, respectively. Log rank test *P* 0.037.

After a median follow-up of 52 months (IQR 20.8 – 57.7), 25 patients reached the combined primary endpoint, including 3 fatal and 22 non-fatal cardiovascular events (Table 4). Patients were censored at start of renal replacement therapy (*n* = 45), death other than cardiovascular (*n* = 15), loss to follow-up (*n* = 21), and at the end of the study observation period. Cumulative incidence of the primary endpoint in tertiles of urinary excretion of PCS was estimated with the Kaplan-Meier method (Figure 2). Log rank test was significant for differences between these 3 groups (*P* 0.037). In univariate Cox proportional hazard analysis, 24 h urinary excretion of PCS was directly associated with cardiovascular disease during follow-up (Hazard ratio (HR) per 100 μmol increase 1.112, *P* 0.002, HR highest vs. lowest tertile 3.011, *P* 0.03). Other significant variables include age (HR 1.064, *P* 0.002), systolic blood pressure (HR 1.021, *P* 0.04), prior cardiovascular disease (HR 5.880, *P* < 0.0001), presence of diabetes mellitus (HR 4.420, *P* 0.0003), albumin (HR 0.872, *P* 0.0003), eGFR (HR 0.973, *P* 0.02), 24 h proteinuria (Ln) (HR 1.298, *P* 0.009). We then built different multivariate models, each consisting of 3 variables (24 h urinary excretion of PCS and 2 other variables) (Table 5). In each model 24 h urinary excretion of PCS remained a significant predictor of cardiovascular events during follow-up. We also built sequential models with addition of variables that were considered confounders a priori, i.e., age, presence of diabetes mellitus, protein intake and eGFR. Again, 24 h urinary excretion of PCS remained associated with cardiovascular events during follow-up (HR 1.103 (1.006 – 1.209), *P* 0.04).

**Table 4:** Cardiovascular events

**Events (n = 25)**	**N (%)**
*Non-fatal*	22 (88%)
Cardiac	9 (36%)
New onset angina, conservative	4 (16%)
New onset angina, invasive	3 (12%)
Acute myocardial infarction	1 (4%)
Ventricular arrhythmia	1 (4%)
Ischemic cerebrovascular accident	2 (8%)
Peripheral arterial disease	11 (44%)
*Fatal*	3 (12%)

**Table 5:** Cox proportional hazard multivariate models of time to first cardiovascular event (number of events = 25)

**Model**	**24 h urinary excretion of *p*-cresyl sulfate**	
	**Hazard ratio per 100 μmol increase (95% confidence interval)**	***P***
1. Unadjusted	1.112 (1.041 – 1.187)	0.002
2. Prior cardiovascular disease and diabetes mellitus	1.083 (1.005 – 1.167)	0.04
3. eGFR and 24 h proteinuria (Ln)	1.120 (1.042 – 1.205)	0.002
4. Creatinine and age	1.120 (1.040 – 1.206)	0.003
5. Albumine and body mass index	1.078 (1.014 – 1.146)	0.02
6. Systolic blood pressure and parathormone (Ln)	1.106 (1.038 – 1.179)	0.002
7. Hemoglobin and C-reactive protein (Ln)*	1.112 (1.041 – 1.187)	0.002

*Hemoglobin was entered as time-dependent covariate.

We also explored the relationship between 24 h urinary excretion of PCS and overall mortality, as well as renal disease progression. In this cohort, we observed a total of 21 deaths (5 cardiovascular, 7 oncologic, 1 infectious and 8 other deaths), again censored at start of renal replacement therapy and loss to follow-up. In univariate Cox proportional hazard analysis, 24 h urinary excretion of PCS was directly associated with overall mortality (HR per 100 μmol increase 1.090, *P* 0.02). Other significant variables include age (HR 1.072, *P* 0.0003), diabetes mellitus (HR 4.011, *P* 0.002), eGFR (HR 0.966, *P* 0.01), systolic blood pressure (HR 1.025, *P* 0.02), hemoglobin (HR 0.743, *P* 0.02), C-reactive protein (Ln) (HR 1.532, *P* 0.02) and PTH (Ln) (HR 1.680, *P* 0.01). In addition, we built different multivariate models with 24 h urinary excretion of PCS remaining a significant determinant of overall mortality in all trivariate-adjusted models (Table 6). As for cardiovascular disease, we also built sequential models with addition of variables that were considered confounders a priori, i.e., age, presence of diabetes mellitus, protein intake and eGFR. Again, there was a clear, albeit non-significant, relationship between 24 h urinary excretion of PCS and overall mortality (HR 1.098 (0.987 – 1.222), *P* 0.09).

**Table 6:** Cox proportional hazard multivariate models of time to death (number of events = 21)

**Model**	**24 h urinary excretion of *p*-cresyl sulfate**	
	**Hazard ratio per 100 μmol increase (95% confidence interval)**	***P***
1. Unadjusted	1.090 (1.015 – 1.169)	0.02
2. Age and diabetes mellitus	1.102 (1.015 – 1.198)	0.02
3. eGFR and albumine	1.090 (1.004 – 1.182)	0.04
4. Systolic blood pressure and gender	1.082 (1.010 – 1.158)	0.03
5. Hemoglobin and C-reactive protein (Ln)	1.083 (1.005 – 1.167)	0.04
6. Calcium and parathormone (Ln)	1.092 (1.009 – 1.182)	0.03

Progression of renal disease was observed in 55 patients during follow-up. However, there was no association between 24 h urinary excretion of PCS and renal disease progression (*P* 0.19).

## Discussion

We studied the clinical determinants of intestinal uptake of *p*-cresol in a cohort of patients with different stages of CKD, and explored its relationship with cardiovascular disease, overall mortality and renal disease progression. Assuming steady state conditions, 24 h urinary excretion of PCS was used to estimate intestinal uptake of *p*-cresol. The key findings are as follows: (i) intestinal uptake of *p*-cresol is highly variable in CKD patients, although factors determining this interindividual variability remain largely unknown; (ii) intestinal uptake of *p*-cresol is associated with cardiovascular disease and overall mortality during follow-up, but not with renal disease progression.

Serum PCS has been linked to cardiovascular disease [3-5], overall mortality [6,7] and progression of CKD [8]. Intriguingly, there is a wide dispersion of individual serum concentrations of PCS in CKD patients, even after adjustment for renal function loss [3,7,11]. Recently, our group identified intestinal uptake of *p*-cresol to be a main determinant of this interindividual variability [12]. Therefore, we hypothesized that intestinal uptake of *p*-cresol, in addition to diminished clearance of PCS due to renal failure, is directly associated with adverse outcomes in CKD.

In this cohort we observed a direct relationship between intestinal uptake of *p*-cresol and cardiovascular disease during follow-up, as well as overall mortality. These associations remained significant after adjustment for several risk factors, including markers of renal (dys)function. This observation points to the large intestine as a unique and important contributor to adverse outcomes in patients with CKD, demonstrating the complexity of the gut-kidney axis [18].

As it was already demonstrated that serum PCS was related to CKD progression [8], we hypothesized that intestinal uptake of *p*-cresol, as a marker for the renal tubular load of PCS, was also an independent determinant of renal function deterioration. At odds with the findings of Wu *et al.*, we were not able to demonstrate an association between intestinal uptake of *p*-cresol and renal disease progression. This questions the true relevance of PCS in the pathogenesis of CKD progression, especially as serum PCS was neither related to CKD progression in our original cohort (unpublished data), nor are we aware of clinical data confirming this association in another patient group. We can, however, not exclude the possible impact of genetic or environmental differences as our cohort mainly consists of Caucasian patients in contrast to the Asian patient group studied by Wu *et al.*[8].

As intestinal uptake of *p*-cresol appeared highly variable, we explored factors determining this interindividual variability. Protein intake was the most significant determinant of intestinal uptake of *p*-cresol. Age, body mass index and hemoglobin were other determining factors. Presence of diabetes mellitus was also independently associated with higher intestinal uptake of *p*-cresol, which confirms and extends previous observations of a relationship between higher serum concentrations of PCS and diabetes mellitus [4,19].

However, it must be noted that most of variability of intestinal uptake of *p*-cresol could not be explained by standard demographic and biochemical variables (model R^2^ 0.22). Intestinal uptake of *p*-cresol is a composite of its intestinal generation and intestinal disposition. Intestinal generation of *p*-cresol depends on colonic microbial fermentation of tyrosine. Factors governing this fermentation process are still largely unknown. It is assumed that an important regulator of bacterial metabolism is substrate availability, especially the ratio of available fermentable carbohydrate to nitrogen (protein) [20,21]. Accordingly, protein intake was a main determinant of intestinal uptake of *p*-cresol in our study [22]. In addition, it is suggested that CKD goes along with a different microbial composition [23]. Therefore, CKD might also have an impact on the microbial metabolism as such, but this needs further examination.

Intestinal disposition of *p*-cresol is the second factor determining intestinal uptake of *p*-cresol. Pharmacokinetics of *p*-cresol in health and renal disease remain to be elucidated, as are possible interindividual differences in intestinal *p*-cresol disposition.

There are limitations to our study. First, we estimated total intestinal uptake of *p*-cresol by 24 h urinary excretion of PCS, thus assuming negligible non-renal clearance of serum PCS and minor conjugation to other metabolites (i.e., *p*-cresyl glucuronide). Second, due to the relatively low number of events, full multivariate adjustment was not possible. To overcome this limitation, we have built multiple trivariate models, consistently demonstrating the predictive performance of intestinal uptake of *p*-cresol. Third, our study population mainly consists of patients of Caucasian origin. Care must be taken to extrapolate our data to other patient populations. Finally, assessment of completeness of urinary collections is arbitrary. We assumed completeness of urinary collections when urinary excretion of creatinine was within 2 standard deviations of the mean creatinine excretion for the geographical region of this study (INTERSALT study [15]).

## Conclusions

Intestinal uptake of *p*-cresol demonstrates substantial interindividual variability, but associates with cardiovascular disease and overall mortality in patients with CKD. Insights into mechanisms governing intestinal generation and absorption of *p*-cresol may lead to identification of novel therapeutic targets to reduce cardiovascular disease and overall mortality in CKD.

## Abbreviations

CKD: Chronic kidney disease; PCS: *p*-cresyl sulfate; PTH: Parathyroid hormone; HR: Hazard ratio.

## Competing interests

The authors declare that they have no competing interests.

## Authors’ contributions

RP conceived of the study, collected and interpreted the data, performed the statistical analysis, and drafted the manuscript. LV collected and interpreted the data, and helped to draft the manuscript. KV interpreted the data, and revised the manuscript. PA interpreted the data, and revised the manuscript. BB collected and interpreted the data, and revised the manuscript. KC collected and interpreted the data, and revised the manuscript. DK collected and interpreted the data, and revised the manuscript. PE collected and interpreted the data, and helped to draft the manuscript. BM conceived of the study, collected and interpreted the data, performed the statistical analysis, and drafted the manuscript. All authors read and approved the final manuscript.

## Pre-publication history

The pre-publication history for this paper can be accessed here:

http://www.biomedcentral.com/1471-2369/15/87/prepub
